# Broken Minds and Beaten Bodies: Cultures of Harm and the Management of Mental Illness in Mid- to Late Nineteenth-century English and Irish Prisons

**DOI:** 10.1093/shm/hky038

**Published:** 2018-06-15

**Authors:** Catherine Cox, Hilary Marland

**Affiliations:** 1 Centre for the History of Medicine of Ireland, School of History, University College Dublin, Newman Building, Belfield, Dublin 4, Ireland; 2 Centre for the History of Medicine, Department of History, University of Warwick, Coventry, UK

**Keywords:** prison, separate confinement, mental illness, harm, punish

## Abstract

This article explores the relationship between the prison and mental illness, focusing on the ways in which the system of separate confinement was associated with mental breakdown and how maintaining the integrity of prison discipline mitigated against prisoners obtaining treatment or removal to an asylum. Examples are taken from English and Irish prisons, from the introduction of separate confinement at Pentonville Prison in London in 1842 until the late nineteenth century, exploring the persistence of the system of separation in the face of evidence that it was harming the minds of prisoners. The article also briefly examines the ways in which prison doctors argued that they were dealing with special categories of prisoner, adept at feigning, intrinsically weak-minded and whose mental deterioration was embedded in their criminality, factors that served to reinforce the harmful environment for mentally ill prisoners.

In January 1846 Pentonville’s Medical Officer, Dr G. Owen Rees, made the following entries in his journal describing the state of health of several of his prisoner patients during the previous month:

That reg. no. 700 shows symptoms of hallucination, stating the warders poison his food, & believes the medical men know it, but wish to kill him to examine his body; - That, Convict John Vincent is convalescent, Prisoner Thos. Sharpe reg. no. 700 is worse & looks ill … had not yet been removed to the Infirmary, fearing it might tend to excite him, he refuses to take his medicines & had recommended that the said Prisoner should be walked about the grounds with an attendant; That, he had read a second letter received by the Governor concerning Convict Wm. Hancocks, reg. no. 1023, which states that his reason was totally upset for 12 months; - That Convict John Grundle Reg. No. 776 appears excited on religious subjects, & had been placed under treatment accordingly … that Pris. John Javis Reg. No. 865 is very anxious, believing he has been pardoned, an impression he derived from the visits of the Chaplain having been more frequent that usual …^1^

While Convict John Vincent (convict no. 578), ‘the subject of mental disease’, was later reported convalescent, the situation regarding Thomas Sharp deteriorated further, when he attacked Pentonville’s gardener on 15 January with a basket-maker’s knife that ‘he had secreted for the purpose’ while taking exercise. Sharp was declared to be suffering from mania and was removed to Pentonville’s infirmary, ‘every precaution being taken for his security’.^2^ He was removed to Bethlem Hospital on 20 January.^3^

Sharp’s transfer to Bethlem was almost certainly prompted by his ‘having attempted the life’ of the gardener, and the speed of his removal was uncharacteristically rapid in comparison with other cases recorded in Pentonville’s minute books and journals. As this article will illustrate, other convicts with suspected or diagnosed insanity were retained within the walls of Pentonville for many months or even for the entire duration of their sentence prior to transportation. For some this involved a relaxation of the discipline (as in the case of Sharp who had been allowed to walk in the garden, a concession the prison officers subsequently no doubt regretted) or removal to the infirmary for treatment, while a small number of prisoners were medically discharged. However, other prisoners, even after showing prolonged and severe symptoms of mental disorder, were moved to invalid hulks or to other prisons, or simply retained in their cells. Convict John Vincent was taken to Pentonville’s infirmary, not apparently in an attempt to cure him, but in preparation for his embarkation to Australia on the next available vessel.^4^ In April the Medical Officer reported that Vincent had claimed that he would not be sent abroad, ‘this allusion to his hallucination, he [the MO] believes is made with a view of avoiding transportation’.^5^ No more is heard of Vincent who was presumably transported shortly afterwards. These examples indicate the frequency of mental disorder amongst Pentonville’s prisoners, incidences of which were noted month after month in the prison’s minute books and journals by its medical officers, chaplains, governor, warders and schoolmasters. While ever alert to the possibility of shamming and eager to weed such cases out and punish them, the institution’s officers regularly reported instances of mental breakdown that subverted the original intention of the prison, to establish a regime of order and intense regulation that would reform, redeem and improve prisoners’ minds.

In this article we examine the impact of the disciplinary regimes of English and Irish prisons on the mental health of, mostly male, prison inmates in the mid- to late nineteenth century. The new ‘Philadelphia’ or separate system of confinement was introduced at Pentonville Prison in London in 1842 and subsequently at prisons throughout England and Ireland. Support for the separate system—despite concerns about its impact on the mind—was to shape and dominate prison disciplines and philosophies throughout the nineteenth and into the early twentieth century. Not all incidences of mental disorder were prompted by the separate system but many prisoners, already suffering from mental ill health on admission, deteriorated under the inflexible regime it imposed, in particular the long hours of cellular confinement. Effective treatment was hampered by the rigid discipline and the reluctance of the prison officers to fully acknowledge prisoners’ mental breakdown and by the tendency for mentally disordered prisoners to be retained in prison.

Our exploration of English and Irish prisons enables examination of the translation of the Pentonville model within England and to Ireland, at a time when the purest form of the regime at Pentonville was being modified largely because of its association with mental breakdown.^6^ There was much reflection and debate on the part of prison administrators on the advantages and drawbacks of separation and variation in its implementation. Nonetheless support for the separate system, at the core of prison regimes in both contexts, proved remarkably resilient. We ask how far the management of mental illness in prison aimed at mitigating harm to the institution rather than relieving the prisoner patient. We consider how the concerns and interventions of prison doctors and chaplains, shaped and constrained by dual loyalty to the prison system and to the health and well-being of their prisoner patients, contributed to a culture of harm in prisons. We also explore how far evolving claims to expertise and specialist knowledge among prison medical officers in identifying ‘real’ cases of insanity embedded prisoners’ mental health problems in their criminal activities and diminished prisoners’ access to therapeutic care outside prison.

The examples of prisoners’ experiences of mental ill health detailed above amplify the importance of careful reading of the archival sources alongside the published official reports. Only one case of mania, that of Thomas Sharp, was acknowledged by the Pentonville Commissioners in their Annual Report for 1846, along with just five cases of mental delusion.^7^ However, the prison’s minute books and journals reveal that instances of mental disorder—and the ensuing disruption caused to the institution—to be very much higher. As exemplified in the opening extract, in just one month of 1846, the prison surgeon identified five prisoners who were showing symptoms of delusion, confusion, anxiety, excitement or unreason. Over the year 1846 the minute books noted 26 cases where prisoners were described in similar terms or as nervous, incoherent and depressed in spirits, although four of these, it was claimed, were simulating mental disease, dissembling or ‘incorrigible’.^8^ A close survey of institutional records also reveals that prisoners’ mental deterioration was increasingly related to prison discipline and the conditions of separate confinement by those working within the prison system itself. At the same time, prison administrators were keen to preserve the integrity of the system and to moderate any criticism of it, arguing that prisoners’ mental disorder provided further evidence of their criminality, inherent mental weakness and low moral character.

Our findings are at variance with the recent conclusions of Ian O’Donnell who has argued, based largely on his analysis of official documentation and a selection of first-hand accounts, that separate confinement in general did not lead to mental breakdown, and that ‘a fortunate few’ were even fortified by the experience.^9^ Relying on the summaries presented to the Commissioners in Lunacy by Pentonville prison staff, notably the chaplains who were key advocates of the separate system, O’Donnell emphasises, for example, the simulation of mental illness on the part of prisoners adept by means of their criminal activities at subterfuge.^10^ Our examination of Pentonville’s journals and minutes, however, demonstrates that many prisoners initially believed to be feigning came to be recognised as suffering from mental illness by prison staff, and, as the figures for 1846 cited above testify, the number of prisoners suspected of feigning madness was small compared to the number of reported cases of mental disorder.

In taking an approach that investigates under-utilised minute books, correspondence and journals for English and Irish prisons in conjunction with official publications and reports, we aim—rather than reprising their arguments—to put to an empirical test the conclusions of influential studies of the prison, particularly those of Michel Foucault, Michael Ignatieff and David Garland.^11^ These authors have emphasised the imposition of penal power in nineteenth-century prisons and the ways in which new categories were produced in prisons through the discourses of the locally powerful. As psychiatry and medicine expanded its influence beyond nineteenth-century lunatic asylums, prisons became sites of intervention and ‘mental disorders provided ways of constructing social deviance’ and blurred ‘the lines between … medicine and … the jurisdiction of other authoritative bodies’.^12^ Here we aim to explore the complex exercises of authority and decision-making within prisons—notably between chaplains and prison medical officers, key brokers in gauging and responding to mental illness in prison—and how they were shaped by pragmatism and the desire for effective management, as well as by claims to authority and knowledge.

## Separate Confinement at Pentonville ‘Model’ Prison

Efforts ‘to devise a perfectly rational and reformative mode of imprisonment’, starting with the initiatives of John Howard and other reformers, and continuing through the late eighteenth and early nineteenth centuries, culminated in England with the establishment of Pentonville ‘Model’ Prison in 1842.^13^ While Howard and other prison reformers had criticised eighteenth-century prisons for incarcerating the insane, who they then failed to treat, and for the impact this had on other inmates in conditions that were often chaotic and overcrowded, their advocacy of perfectly regulated environments and heightened surveillance in prisons, may well have played a role in reinforcing the relationship between prison confinement and mental disorder.^14^ At Pentonville an ‘experimental’ prison discipline, the separate system, was put into practice in a ‘pure’ and exacting form, aiming to produce the ‘moral transformation’ of prisoners ‘by carefully controlling time, space and bodies’, and the prisoners’ minds.^15^

The rigorous nature of the Pentonville system was largely attributable to William Crawford, founder member and secretary of the Society for the Improvement of Prison Discipline and Reformation of Juvenile Offenders, who had visited Eastern State Penitentiary in Philadelphia and been captivated by the separate system in operation there, and Reverend William Whitworth Russell, who had served as chaplain to Millbank Prison, where he had strongly asserted the authority of the chaplain and the potential of cell visitations in prompting reflection and reform.^16^ Both exulted cellular solitude and emphasised the faculty of prisoners to be redeemed through individual, spiritual reflection, in a system designed to produce a better man, with better morals and a better mind. The system was also intended to ‘truly humble’ and ‘break the prisoner down by solitude’.^17^ Russell’s evidence on cellular confinement to Select Committees on prison reform in 1831 and 1835 and the appointment of Russell and Crawford to the influential positions of prison inspectors for London in 1835 enabled them to steer the Home Secretary towards acceptance of a stringent form of separate system at Pentonville.

This was in spite of the opposition of numerous critical voices who warned that separate confinement would put at risk the minds of prisoners, including *The Times* newspaper, novelist Charles Dickens and prison governors, such as George Laval Chesterton, Governor of Cold Bath Fields Prison who supported the rival silent system in combination with associated labour.^18^ Chesterton claimed that, while prisoners were liable to attempt suicide given the ‘bitter griefs’ that ‘assailed their minds’ in prison, in over 20 years there had been no successful attempts in his own institution, due to close watching and the support and encouragement offered to prisoners in a desponding state of mind.^19^ He also argued that many criminals were habitual offenders, incapable of reform and that the separate system was unnecessarily cruel, resulting in ‘mental depression’ and ‘direful torture’; any reform it produced would be only temporary.^20^ While it was broadly accepted that all prisons tended to harbour mentally ill prisoners, the separate system was charged with the actual production of mental distress. The evidence was compelling. Dickens who had visited Eastern State Penitentiary, the model for Pentonville, where he had spoken to some of the inmates, famously decried the separate system as ‘cruel and wrong’, ‘this slow and daily tampering with the mysteries of the brain’.^21^ Britain’s first government prison, Millbank Penitentiary, which opened in 1816, saw inmates’ death rates increase markedly when separate confinement was enforced in 1837, and in July 1841 the system was modified. Its medical officer, Dr Baly, warned that prisoners needed to be carefully watched, given the risks of separate confinement.^22^ The prohibition of contact during the probationary period was reduced to the first three months at Millbank, after which prisoners were permitted to converse during exercise.^23^ Yet despite evidence revealing the risks of isolation to the mental well-being of prisoners and the concern expressed by officers working in other prisons, Crawford and Russell prevailed, and, as the most influential members of Pentonville’s Board of eleven Commissioners, exerted a powerful influence on Pentonville’s vision and governance, devising the prison’s rules and regime, and handpicking prisoners fit to undergo the rigours of separation.

The Pentonville regime confined prisoners in their cells, where they worked, ate and slept, alone and in silence for 23 hours a day during an 18-month probationary period prior to transportation. During this time the convicts’ day was mapped out in meticulous detail.^24^ Outside of their cells, as they were moved through the prison to exercise or to attend chapel, prisoners’ faces were hooded, making it impossible for them to recognise each other. In chapel they sat in separate closed stalls.^25^ Punishment for infringing rules led to confinement in the dark cells on a bread and water diet, or to beatings with the birch or the cat for more severe offences, such as assaulting warders. Such penalties were also likely to be inflicted on those suspected of feigning mental disorder. The Pentonville regime of separate confinement was recognised as being ‘taxing’ to its inmates carefully selected by Crawford and Russell, who were to be aged between 18 and 35 years, in good health, and first time offenders. The regime was also considered more suited to men. When Brixton and Mountjoy women’s prisons were opened in the 1850s, female convicts would be placed for shorter periods in separate confinement, reflecting perceptions of the ‘essential differences between the mental and physical conditions of the sexes’.^26^ Women prisoners were viewed as difficult and disruptive, and also less able to withstand the rigour of prolonged separation. Meanwhile, at Pentonville while many male convicts appear to have settled into the routine and ‘bided their time’, showing no apparent signs of damage, others were affected by the silence and solitude.^27^ The dangers of the system to the minds of prisoners and the high risk of mental breakdown was acknowledged in the Pentonville Prison Act (1842), which specified that prisoners who showed signs of mental illness were to be reported to the Secretary of State and transferred to an asylum. Meanwhile, the prison’s regulations underlined the importance of vigilant observation by both chaplain and surgeon of the ‘*state of mind* of every prisoner’.^28^

Yet, supporters of the separate system insisted that the minds of prisoners at Pentonville would be ‘improved’, by means of teaching, preaching and individual cell encounters. Their optimism seems to have been at one and the same time genuine and unfounded. In detailing the regime at Pentonville, Crawford and Russell explained:

In the stillness and solitude of his cell the long-forgotten religious precept, the early domestic admonition and example, the last solemn warning of the dying parent, the traces early impressed upon the youthful memory, all arise before the guilty conscience with a vividness and force to which the solemnity of the occasion gives tremendous emphasis. All artificial supports are now withdrawn, and the culprit is made to feel the reality of his condition, and the fearfulness of that prospect leads him to think of ‘righteousness, temperance, and judgment to come’.^29^

It was claimed that opportunities for contact with staff and regular cell visits, particularly those of the chaplains who were to be key figures in urging repentence, reform and improvement, would safeguard prisoners’ minds and differentiated the Pentonville regime from Eastern State Penitentiary with its more restricted access to prison staff.^30^ Schooling, moderated by the chaplains who took charge of the schoolmasters and curriculum, was intended to stimulate the mental faculties and offer the prisoners better life skills following transportation.

The most important component of the regime, however, at least in the eyes of the chaplains, was the cell visitation, intended to draw out true declarations of repentence and redemption on the part of the convicts. Reverend Kingsmill at Pentonville was diligent in this duty; his journal demonstrates his commitment and the long hours he devoted to his work. In the Commissioners Report for 1846 Kingsmill asserted that the influence of Pentonville’s discipline on the mind was, for the majority of prisoners, ‘of a most beneficial character’. Most prisoners applied themselves industriously to ‘mental improvement’, and only those with a predispositon to sullenness, resistence to learning or unable to take advantage of it, or those whose minds were preoccupied with one subject, were at risk under the separate system.^31^ Yet in the first months after Pentonville opened its prisoners began to show symptoms of mania, depression, delusion, hallucination, panic, anxiety and morbid feelings, or were noted to be ‘irritable’ or ‘sullen’. Such symptoms were described, debated and at times disputed by the prison’s officers, as they tested the viability of the regime and the reputation of the prison, its administrators and advocates of spiritual reform. Kingsmill’s own optimism was to turn to doubt when confronted with so many cases of mental breakdown. Initially in his journal entries, in contrast to his reports to the Commissioners, Kingsmill questioned certain components of the regime, claiming in 1846 that the restricted diet and the ‘additional depression’ of the dark cell ‘have a tendency to produce a species of decline, of which the opposite treatment becomes the best remedy’. However, he was also ‘happy to record his great satisfaction at a manifest progress towards an ameliorated use of punishment in the Prison’.^32^ By 1849 even his official report to the Commissioners concluded that the value of separate confinement ‘in a moral point of view has been greatly over-rated’, although he believed it still offered the opportunity for reflection and awaking the conscience of prisoners.^33^

The Pentonville minute books illuminate in detail the messy and protracted process of agreeing diagnoses and the absence of a clear procedure to deal with suspected cases of mental breakdown. The case of convict no. 969, Joseph Lees was brought to the attention of the governor and medical officer, Rees, by Chaplain Kingsmill in March 1846. He described Lees as ‘being much depressed & strange in his manner, & remarks that his disposition is very bad, & marked by a great deal of low cunning’. Dr Rees was unconvinced, insisting that Lees ‘is better, and that his mental symptoms are decidedly shammed, & that, the Prisoner does not deny the fact, when accused’.^34^ His case came up again, in June 1846, when the assistant schoolmaster reported him to the governor, medical officer and chaplain, as ‘wholly unfit for the discipline of the Prison’ following remarks the convict had made suggesting ‘he is either out of his mind or, pretending to be so’. Rees was dismissive, insisting that ‘this man began to impose immediately after experiencing kind treatment in March’.^35^ By September Rees was anxious to consult his colleague Dr Seymour on the case, as Lees had convinced ‘his friends’ that he was insane though Rees was ‘still inclined to consider him as imposing’.^36^ Seymour confirmed Rees’ opinion but Lees’ case continued to be debated. Lees’ uncle who visited his nephew in prison stated that he had always been eccentric, a thief from childhood and had a ‘jeering impertient way of conducting himself’, but that he was not out of his senses. His mother, who had also recently visited him, thought that he was.^37^ In January 1847, Lees spoke to Major Joshua Jebb, Surveyor-General of Prisons and one of Pentonville’s Visitors, in the presence of the surgeon ‘in [a] most extraordinary & incoherent manner’. The day after he was visited by the Medical Commissioner, Dr Ferguson, who described him as ‘not incoherent but extremely impudent’.^38^ The case dragged on and in March 1847, a year after attention had first been brought to Lees’ case, Seymour certified Lees as sane and he was transferred to Millbank in anticipation of transportation.^39^ However, it appears he never departed. In February 1849 Lees was one of three prisoners whose cases were brought to the attention of the Pentonville Comissioners by the chaplain as ‘men about whose soundness of mind they entertained serious doubt, but who were subsequently punished repeatedly for prison offences, were removed in the 3^rd^ or incorrigible class and are now in Bethlehem’.^40^

This case again illuminates how records of the day-to-day running of Pentonville contrast starkly with the limited attention played to mental illness in the Commissioners’ published reports.^41^ Cases such as Lees tended not to be discussed in detail or at all in official accounts or to appear in lists of prisoners declared insane, despite the work and uncertainty they produced for the institution’s officers. Yet while Pentonville’s officers resisted removals to Bethlem Lunatic Asylum, the steady accumulation of prisoners there was noted by others. By 1850 John Webster, consulting physician to St George and St James’ Dispensary, praised Pentonville and Millbank prisons for improving prisoners’ physical health, yet noted that ‘61 prisoners were sent to Bethlehem Hospital [from both prisons] during the last ten years, who had become insane; 47 being men, and 14 women; besides four men who came from the hulks, but had previously resided in Pentonville prison’.^42^

While other commentators, including its President Peter Laurie, grumbled that Bethlem became the repository for prisoners unable to withstand the Pentonville regime and a signifier of the failure of separate confinement, still many mentally disturbed prisoners were retained in the prison or ended up being moved to other prisons, notably Millbank, or to the prison hulks.^43^ The eagerness of Pentonville’s officers and Commissioners to limit removals to asylums can be explained, in part at least, by the officers’ efforts to protect and defend the system of discipline. The use of creative labelling is likely to have also played a role in reducing the number of removals to Bethlem, which was largely limited to what was referred to as cases of ‘real insanity’. Generally removals to Bethlem were labelled as suffering from insanity or mania, while other categories of mental disorder were retained in Pentonville. Prison staff produced their own descriptors of mental afflictions, using a range of labels not normally seen in other contexts, including asylum practice. This unique taxonomy manifested itself—in Pentonville and other prisons—in expressions such as ‘sullen’ or ‘sullen obstinacy’, ‘strange combinations of cunning and weakness’, ‘knavery and almost imbecility of mind’, ‘morose’, ‘eccentric’, ‘excitable’ ‘mischievous’ and ‘irritable’.^44^

In 1847 Pentonville’s Commissioners were at pains to show that only five cases of insanity had been removed to Bethlem over four years of operation, suggesting that this was less than for other institutions of confinement. However, in addition to those ‘who were actually insane’, 12 other convicts had ‘exhibited symptoms of original weakness of intellect, or have laboured under partial delusions’. Of these, three had been removed to the invalid hulk, and nine had recovered at Pentonville on being given a more stimulating diet and employed in out-of-door labour.^45^ Again in 1851 just two removals of cases of ‘insanity’ to Bethlem were recorded, but in addition three cases of delusion were noted, two of whom were said to have recovered in prison while the other prisoner was removed as ‘unfit for further separate confinement’:

Besides the cases of insanity and delusion … there were observed other cases, in which mental depression, irritibility, natural feebleness of intellect, and other conditions not amounting to actual disease, existed, and rendered relaxation or suspension of the discipline, necessary in the first place, and removal from the prison advisable at a subsequent period. They amounted in all to 22.^46^

By the early 1850s, it was not only Chaplain Kingsmill who was expressing concern about the impact of the regime. After the sudden deaths of Crawford and Russell in 1847, efforts were made to tone it down, and the length of separation in Pentonville reduced. By 1848 it had been cut to 12 months and again to nine months by 1853. Major Jebb had noted his doubts about persevering with efforts to retain ‘mental cases’ in Pentonville at the start of 1847 when six convicts had been placed in the ‘Garden Class’ in an attempt to relieve their symptoms. Rather, he argued, such men ought to be removed at once, and their places taken by others ‘more able to bear the discipline’.^47^

While regarded as a ‘failed experiment’, one that had been played out very much in the public eye, committment to the system of separation remained evident, for example, in the 1850 Select Committee on Prison Discipline, chaired by Home Secretary Sir George Grey, whose members resolved that ‘entire separation, except during the hours of labour and of religious worship and instruction, is absolutely necessary for preventing contamination, and for securing a proper system of prison discipline’. The Committee also characterised the variation in prison construction and discipline, then evident throughout England, as a ‘great evil’. Despite hearing ‘conflicting’ evidence in terms of the result of the separate system at Pentonville, Grey’s committee endorsed its uniform application in prisons across England.^48^ Miles Ogborn contends that this persistence in support for the separate system and its continued influence over penal policy, despite concerns about its impact on prisoners’ minds, reflected the Victorian infatuation with ‘uniformity’ and with disciplinary rationality that eliminated ‘inequality, uncertainty and inefficiency of punishment’, deemed to be unfair to ratepayers and prisoners alike.^49^ By 1850 Pentonville had inspired ten new prisons to be built on the same model, and ten others had converted to the separate system in England.^50^ Many prisons continued to enforce separation though for the most part using modified versions of the Pentonville regime—Irish prisons, as we will see, were particularly interesting in this respect—resisting mounting concern about its impact on the mind.

## Beyond Pentonville: Embedding the Separate System

Implementation of the separate system, the aim of most prisons, was, however, in effect patchy during the second half of the nineteenth century. This was often due to overcrowding, resulting in over-stretched prison staff and prisoners doubling up in cells, which did away with the principal of cellular isolation. At Liverpool Borough Gaol, specifically designed for the separate system and the largest local prison in England when it opened in 1855, the governors dealt with overcrowding by periodically ending cellular isolation, allowing two or three prisoners to sleep in a cell. Dealing with overcrowding in the prison, alongside monitoring the impact of more punitive prison rules—introduced from 1864 onwards—on prisoners’ minds increased the burden on the medical officers and chaplains who were expected to assess each prisoner’s physical and mental fitness to undergo the regime.^51^ In 1856 a parliamentary review of the disciplinary systems in operation in English and Irish prisons found that many, including Reading, Bedford, Bath, Mountjoy and Pentonville, ‘fully carried out’ the separate system, while in others implementation was partial or completely absent.^52^ Reading Gaol removed small numbers of county prisoners considered insane to Littlemore Asylum in Oxford (regretting the cost to the county of the extra diet and attendance this entailed) and convicts to Bethlem, but, despite steady reporting of mental breakdown amongst the gaol’s inmates, the faith of both the prison chaplain and surgeon at Reading in the separate system was unwavering. ‘Lunatic Patients’ requiring ‘relaxation of the discipline of separate confinement’ were taken on a regular basis into the infirmary with a view to returning them to complete their sentences under the full rigour of separation once recovered,^53^ and in 1853 Reverend John Field declared to the Visiting Justices that

I am more than ever convinced that you have adopted a system of prison discipline the most corrective that human wisdom has ever devised. I should therefore deeply lament both for the sake of Society and the Souls of Men that it should be subverted & abandoned, or even be modified as to impair its efficiency.^54^

However, other medical officers, prison governors and chaplains were apprehensive about the separate system of discipline and its risk to mental health. Already by 1847 Wakefield’s prison officers were expressing their reservations in a report to the local magistrates, its chaplain concluding that ‘there appeared little doubt that cases of mental delusion might be attributed to the separate system, as they were much benefitted by the adoption of the modification of the discipline, shewing that the system of *total* separation was not universally applicable’. Wakefield quickly introduced modified dietary and exercise regimes, and allowed prisoners more outdoor exercise, which was claimed to reduce their mental stress.^55^ By the early 1850s official reports were casting doubt on the initial evidence that had led to the advocacy of separate confinement. Surveyor-General of Prisons and Pentonville’s architect, Jebb, who had been consistently more guarded in his commitment to the system of separation, suggested in 1852 that Crawford’s enthusiasm after his visit to the Eastern State Penitentiary had been based on ‘slender data’, and that

so far as regards the assertion, that discipline, as enforced at that prison, would have no unfavourable effect on the mind or health, or the inference that it could be safely enforced in the case of all prisoners for lengthened periods, experience has since shown, that the anticipations he entertained have not been altogether realized.^56^

Jebb had been instrumental, however, in extending the separate system to Mountjoy Convict Prison in Dublin, opened in 1850, and designed according to the Pentonville model.^57^ Yet some Irish prison administrators were dubious about the system. Following his visit to Pentonville in 1850 in preparation for the reception of the first prisoners at Mountjoy, the Inspector of Government Prisons in Ireland, Henry M. Hitchins warned the medical officer at Mountjoy, Dr Francis Rynd, that

The objections which have been urged against this [separate] system … appear to be principally directed to the injurious tendency of long periods of separate confinement to produce a general debility of mind and body—this aggravating in the prisoners any previous predisposition which may have existed to the serious classes of diseases which not unfrequently arise from this state of depression, and … which, operating on some kinds, produce imbecility or utter prostration of the mental powers.^58^

While in agreement that ‘“separation should be the principle” upon which Mountjoy Prison is to be conducted’, Hitchins concluded that ‘many details of Pentonville which being *extreme* are necessarily futile, may be safely avoided’. He introduced various modifications at Mountjoy, which he believed ‘did not impair the system’.^59^ Male convicts were placed in separation for eight months, rather than the 12 still imposed in England, and the emphasis placed on religious instruction, stressed at Pentonville, was downplayed; the Mountjoy schoolmaster was to confine his instruction to secular topics and was not directly supervised by the chaplain, as was the case at Pentonville.^60^ Over the next four years, the implementation of the separate system at Mountjoy ‘varied considerably’. For the first 18 months after the opening of the prison, it had been enforced strictly and with rigour, ‘the beneficial results of which are fully attested by all the officers connected with its administration’.^61^ Subsequently, the employment of prisoners in trades assumed greater prominence in the prison’s regime, while the time allocated to religious and secular education was reduced. This system was in turn amended on the recommendation of the 1854 parliamentary inquiry into the management and discipline of Irish convict prisons, prompted by the cessation of transportation and the introduction of penal servitude, when the primary purpose of separate confinement, ‘moral and religious improvement’, was re-asserted.^62^

Individual prison officers shared in the belief that the benefits of the separate system outweighed the disadvantages and the potential danger to prisoners’ minds. Having overseen the ‘strict’ and ‘relaxed’ regime in operation at Mountjoy, Governor Robert Netterville concluded in 1854 that the strict enforcement of the separate system had ‘a much better effect on the prisoners … they were more orderly, respectful, and better behaved’.^63^ The prison’s Church of Ireland Chaplain, Gibson Black, insisted that the ‘*unremitting application*’ of the separate regime, in terms of duration and emphasis on religious and spiritual refection, guarded against the danger of producing insanity.^64^ When the separate system was originally enforced at Pentonville, with its most rigorous form of discipline, Kingsmill’s successor Chaplain John Burt contended, ‘there was scarcely any excess of insanity … since the discipline has been relaxed, and the term reduced, the insanity has increased’.^65^ In his 1852 defence of the separate system, published after the deaths of Crawford and Russell, and the modification of the regime at Pentonville, Burt observed that:

It is to be remembered, that in ordinary life mental disturbance is not unfrequently the consequence of heavy calamities. Under any system of severe punishment, a *degree* of risk to the mind is therefore inevitable. Penal inflications of all misfortunes involve consequences the most disastrous; and the danger which is a natural result of all other great misfortunes, is inseparable from these.^66^

He went on: ‘Pressure upon the mind under a Cellular System is a necessary concomitant of its characteristic excellence’.^67^ To understand the cases of mental breakdown that occurred at Pentonville, Burt contended, it was necessary to make a ‘careful estimate of the extent of insanity, especially of the slighter forms of mental disturbance, in private life, and of the mental peculiarities of the criminal class’.^68^

## Retention in Prison and Harm

High rates of mental distress among prisoners remained a feature of prison life and the mental condition of inmates became a matter of concern almost immediately a new prison was opened or as the separate system was put into practice. Many prisoners were already ill at the start of their sentences before being exposed to separate confinement, and some prisons dealt deftly with the admission of mentally ill prisoners in an effort to maintain the integrity of the prison system and to reduce the workload of prison staff. During his visit to Kirkdale Prison in May 1855, where Chaplain Richard Appleton was a firm advocate of the separate system, prison inspector Herbert P. Voules reported that four prisoners found insane on admission had been transferred to local asylums at Rainhill and Prestwich within a month or six weeks. At Leeds Borough Gaol, which Voules inspected in March 1855, he reported on the attempted suicide of one male prisoner—he had cut his throat with a knife—and the relatively swift transfer of another prisoner to the local asylum. Again, it was concluded that the prisoner was insane on admission.^69^ However, other prisoners were retained when clearly suffering from mental illness, in some cases for many years. Charlotte, aka Creswick, Oakley, was first admitted to Liverpool Gaol in 1888, in the first of several committals. Despite her disturbed and ‘manic’ behaviour she was only removed to Rainhill Asylum in 1893.^70^

Thomas Bourke (convict no. 1742) was aged 15 years when convicted of burglary and robbery in April 1853. As a juvenile, he was first sent to Philipstown Prison, Co. Offaly, an invalid depot and associated labour prison, and then moved to Mountjoy in July 1855, where he was placed on the separate system. From the start, his behaviour was extremely disruptive. He repeatedly broke the rule of silence, assaulted wardens and his fellow prisoners, destroyed his cell, feigned suicide, and refused to work. Frequent punishments—either by placing Bourke on a reduced diet, stopping his meals altogether, or confining him to a dark cell—did not improve his behaviour. He was moved within the system, transferred to the associated labour prison at Spike Island in Co. Cork, back to Mountjoy for a second period in separation, and then back again to Philipstown Prison. He was finally transferred to Dundrum Criminal Lunatic Asylum, Dublin, in 1860 after seven years in various prisons.^71^ At this time, Spike Island was often utilised to take in prisoners, many from Mountjoy, deemed unfit for separation but not categorised as ‘truly’ insane and therefore ineligible for transfer to Dundrum Asylum. This included weak-minded or chaotic and disruptive prisoners like Bourke.

Prison regulations charged medical officers with adjudicating on inmates’ fitness to withstand taxing and strenuous prison regimes, allegedly guarding against the unnecessary infliction of cruelty on physically and mentally ‘weak’ prisoners while simultaneously upholding the ‘punitive’, ‘rule of severity’ and ‘less eligibility’ inherent to prison discipline.^72^ These constraints of ‘dual loyalty’, Joe Sim and others have argued, hampered the ability of prison medicine to work either independently or benevolently.^73^ It produced tensions between doctors’ status as employees of the prison system and their roles in monitoring and approving the disciplinary aspects of the prison regime, and the obligation to care and lobby for the health of their prisoner patients. William Guy, Medical Superintendent of Millbank Prison between 1859 and 1869, and one of the most influential prison doctors of the mid-nineteenth century, exemplified the complexities of the prison medical officers’ role. Guy was an avowed humanitarian, a scientist and advocate of the use of statistical tools to evaluate success. He concluded that the social aim of health needed to be balanced against other social claims, incorporating the Benthamite principles of ‘lenity, severity and economy’.^74^ Guy also supported the use of separate confinement and denied that it destroyed the mind, arguing—in a way not dissimilar to Chaplain Burt—that if damage did ensue this was not the fault of the prison system or physician rather it was a component of the testing but entirely appropriate regime of punishment:

Our system of separate confinement does not appear to affect the mind injuriously. I do not mean to say that a prisoner who comes into prison upon the verge of unsoundness of mind, might not develop into full unsoundness in that time, partly because of the separation; but I am of opinion, also that a prisoner should expect that this may happen to him, and that the possibility of unsoundness must be taken into account as one of the results of his being in prison at all.^75^

Despite these protestations, medical officers, chaplains, governors and other prison staff spent a great deal of time watching over prisoners showing signs of mental distress, investigating cases, organising removals to other institutions, even if delayed, and looking out for signs of shamming. Reaching a consensus as to a prisoner’s mental state was often a protracted process as medical officers sought to differentiate between malingerers, the ‘weak-minded’—a category coming into increasingly common usage by the 1860s—and ‘real’ cases of mental disorder. In his first report as Medical Officer to Millbank Prison in 1859, Guy provided details of nine prisoners—four male and five female—who had attempted suicide. Two were described as ‘serious attempts’ and one of these, a male convict, was sent to an asylum. The remaining cases were dismissed as ‘insincere’ attempts to commit suicide while one was described as a ‘trick to frighten the officer’. Although not specified in the annual report, it is likely these prisoners were punished. An additional 21 male convicts were sent to Bethlem and one to Fisherton asylum, a figure in excess of the 14 certified in the previous year. Of the 22 transferred in 1859, Guy concluded that 16 were insane on reception, four were doubtful cases and two developed insanity while in the prison. He also noted that the increase in the number of insane ‘is to be accounted for by the large number of insane prisoners, who _**…**_ were received from other establishments’, including Pentonville.^76^ Despite insisting that many prisoners were insane on admission, at Millbank transfers to asylums took time. One 40-year-old male convict, admitted in March 1859, whose symptoms of insanity had manifested themselves prior to reception, was not transferred to Bethlem Hospital until June that year.^77^ Other prisons responded by modifying the regime or, as in the case of Bourke, relocated prisoners to other prisons. In December 1860 Mountjoy’s Medical Officer, Dr Robert McDonnell, recommended the removal of two convicts, Andrew McGuirk (convict no. 5331) and James O’Brien (convict no. 5185), to Philipstown Prison, where the separate system was not implemented:

Each of these prisoners has been for some time under special observation and although I hesitate to pronounce either of them as fit subjects for a criminal lunatic asylum yet their marked eccentricity leads me to conclude that they are not fit for the discipline of this prison.^78^

While for McDonnell such cases were not ‘real’ instances of mental disorder and therefore not suited for removal to an asylum, in Mountjoy, they could not be ‘properly met’ or ‘treated’ by repeated and increasingly pointless punishments.^79^ Due to their inherent moral and mental weaknesses, these prisoners had failed the Mountjoy system, unable to improve their behaviour or to benefit from opportunities to reform.

Following the nationalisation of the prison systems in England and Ireland in the late 1870s, prison regimes became harsher and support for cellular separation as a highly efficient and deterrent system strengthened. Interest in imposing rigorous punishment and maximising control overrode the ideals of facilitating reflection and reformation amongst prisoners.^80^ In the same period, medical and psychiatric opinion on the nature and cause of criminality became more penal, and faith in the potential for reform evaporated. Edinburgh psychiatrist, Dr Thomas Laycock argued in 1868 that the habitual/incorrigible criminal was essentially unreformable; a class of people ‘numbering tens of thousands’ so ‘constituted corporeally that they possess no self-control beyond that of an ordinary brute animal …. They are for the most part immoral imbeciles’.^81^ Major Edmund F. Du Cane, Chairman of Directors of Convict Prisons and Inspector-General of Military Prisons, suggested in 1886 that a large proportion of the criminal classes were ‘more or less mentally deficient’, and mocked the ‘burlesque absurdity’ of the evangelical approaches of the reformers of the 1840s.^82^

Prison medical officers argued that they were uniquely placed to identify ‘real’ cases of insanity and differentiate them from those feigning mental disorder and attempting to obtain relief from the rigours of separation or to secure removal to the better conditions experienced in asylums. Such prisoners were regarded as especially problematic and determined in their capacity for deception. While acknowledging that cases of feigning were also found in the military and naval services, Dr John Campbell, Medical Officer at Woking Invalid Prison, noted that among convicts ‘the imposition is carried out with almost incredible determination’.^83^ Prisoners who were considered to have feigned insanity or carried out feigned suicide attempts were usually restrained or punished: whipped, placed on a reduced diet or confined in dark cells. The medical officer at Sligo Prison, Dr Murray, when managing the repeated suicide attempts of prisoner Michael Costello in 1886—he tried to hang himself several times—ordered the ‘Straps & muff to be applied … [the prisoner] to be placed in padded cell and to be visited frequently during the night. To get a cold douche bath twice daily.’ When Costello then refused to eat and speak, Murray commented in his journal: ‘I would wish to have a good powerful Electric Machine supplied to this prison for such cases.’ He tried to force the ‘ruffian’ to eat, using a jaw opener and soft tubes. Costello relented, eating ‘Bread 3 Eggs battered up with 1 quart of milk’. Murray observed in his journal entry that ‘Costello is evidently the worst possible character, but I hope he is now tamed for some time at least’. Although he continued to keep Costello in a padded cell and under observation, Murray remained ‘fully persuaded that his motive was removal to a Lunatic Asylum, where he would have a better chance of escape’.^84^

## Medical Expertise and Professional Boundaries

Dealing with so many cases of mental illness as well as feigned mental disorders, prison medical officers claimed they were developing a particular expertise in detecting shamming as well as framing new categories of mental disorder peculiar to prisoners. This knowledge derived, they argued, from close observation of individual cases within the prison environment, experiences that distinguished them from asylum psychiatrists and medical witnesses. Arthur Griffiths, Deputy Governor at Millbank Prison, contended that prisoners were liable to special and exclusive phases of insanity. These included strange delusions, some featuring allegations of ill-usage and of ‘determined hostility’ on the part of the prison officers and assertions that they heard the officers concocting schemes to do them injury, religious mania, exaggerated destructive tendencies, curious attempts at suicide and persistent feigning often ending in real insanity.^85^ Campbell noted that insane prisoners assumed an ‘ostentatious, over bearing manner, often threatening with violence anyone daring to oppose them’,^86^ while an editorial in the *British Medical Journal* noted that the ‘lunatic convicts’—those sentenced to imprisonment and becoming insane during confinement—are ‘artful and cunning beyond imagination, inciting to insubordination and restlessness those who would otherwise be quiet’. The article estimated that these ‘lunatic convicts’ made up 25 per cent of Broadmoor’s and 10 per cent of Dundrum’s population and should not be allowed to mix with ordinary criminal lunatics as they ‘contaminate and offend’.^87^ By irrevocably linking prisoners’ mental condition to their criminality or their capacity for deception, these categories mitigated against prisoners being treated for insanity or removed to an asylum, and exposed them to harm, whether that be neglect of their cases or punishment.

Tensions arose between prison medical officers and asylum doctors as well as between the Prison Commissioners and Lunacy Commissioners in England and the Directors of Convict Prisons and the Inspectors of Lunacy in Ireland concerning cases that, according to the lunacy officials, had been improperly retained in prison when they should have been removed to an asylum for treatment. While some prisoners were taken to prison infirmaries and offered an enhanced diet, others were left to languish in their cells or removed to padded cells for observation. This was regarded as unacceptable by asylum doctors, who argued that they were best equipped and trained to treat the insane prisoner, and that prisoners who became considerably worse in prison might have fared better if rapidly removed to the specialist care available in the asylum. The treatment of patients in England and Ireland’s expanding asylum systems was shaped throughout the nineteenth century by the principles and practices of moral management which developed regimes based on regularity and order, enhanced diet, work therapy and occupation.^88^ Yet, at the same time, asylums, themselves often overcrowded and struggling to accept new patients and to effectively maintain their regimes of moral management, might be reluctant to take in insane criminals who were regarded as difficult to manage and as a poor influence on other patients.

Prisoners who were removed to asylums were commonly described as being in a terrible state of mental and physical health. Medical staff at Dundrum Criminal Lunatic Asylum and as well as at district lunatic asylums repeatedly complained to the Irish Inspectors of Lunacy and to the prison boards that prisoners arrived from various prisons with serious abrasions, malnourished and in a filthy condition.^89^ In March 1888, a female prisoner, Catherine Kelly, was removed from Tullamore Prison, King’s County to Maryborough District Lunatic Asylum, Queen’s County where she died four days later. Dr Hatchell, the Medical Superintendent at Maryborough, claimed she had been moved to the asylum in a dying state and there were marks and bruises on her body. At the subsequent inquest, Dr George P. O’Farrell, then the medical member of the General Prison Board, concluded that she died of ‘extreme exhaustion’ and that the prison ‘doctor [Dr James Ridley] showed want of judgement in allowing a woman [in such a weak condition] to be removed 18 miles by road’. While Ridley was not accused of wilful neglect, he was criticised by O’Farrell for failing to transfer her to the asylum immediately on reaching a diagnosis of insanity as ‘a few hours often make the greatest difference between safety & danger in the removal of Lunatics’.^90^

The case of Ferdinand Espin Parker, alias Shortlander, similarly centred on the timing of his removal to an asylum. Parker was moved from Shepton Mallet Prison to the Somerset and Bath Lunatic Asylum on 11 June 1885 where he died two days later from a weak heart and ‘prolonged insufficiency of food’. Parker’s attack of insanity was said to have commenced on 19 May, when he was moved to the prison hospital, refusing food and claiming that it had been poisoned, though he was not certified as insane until 5 June. While the Prison Commission argued that the outcome would have been no different if the prisoner had been removed sooner to an asylum, the Commissioners in Lunacy disagreed, claiming that the asylum would have had the expertise in forcible feeding to secure the recovery of the patient.^91^

Serious allegations of actual bodily harm were also made. In December 1897 the *Manchester Evening News* revealed the story of a ‘scandal’ at Strangeways Prison in Manchester, involving Edward Cox, an insane prisoner whose ribs had been broken while he was being restrained. Cox arrived at Prestwich Asylum with severe injuries, but, while the asylum doctors claimed that eight of his ribs had been broken, the officers at Strangeways asserted that all due care had been exercised by the prison medical officers and that ‘only one or two ribs’ were broken. The resulting Home Office inquiry revealed that the prisoner had been admitted to Strangeways in April 1897 and the day before his sentence expired on 9 October he became violently insane. There was a struggle with five prison officers to move Cox to a padded cell, when the injuries occurred. While it was concluded by the Home Office that no unnecessary violence was used, the prison was criticised for its poor standards of medical care and delays in examining Cox.^92^

## Quantifying Harm to Prisoners’ Minds

Alongside disputes over care, the actual rate of mental disorder in the prison system was contested as such figures were made available on a national level for England in the latter decades of the nineteenth century. In 1872, Du Cane speculated that ‘Of the 8,362 men now serving out sentences of penal servitude in England, no less than 252 are absolute lunatic or weak-minded, 308 are subject to bodily infirmities that render them unable to earn a living, and 1,140 are only fit for the lighter kinds of labour, making in all 1,700, or 20 per cent of the whole’.^93^ There was also growing concern in Ireland, with extensive discussion of the rate of insanity in prisons during the hearings of the 1884 Royal Commission on Irish Prisons. In his evidence to the Commission Captain John Barlow, Director of the Irish Convict Prisons, noted that in the previous five years, 34 male and female convicts from Mountjoy Convict Prison and 19 from Spike Island Prison had been sent to Dundrum Criminal Lunatic Asylum. According to Barlow, ‘all the convicts were received in the first instance to Mountjoy’ and he concurred with the view of the commission that ‘the majority became insane during the term of probation [at Mountjoy] rather than after the time when they got to the public works [at Spike island]’. As none of the convicts were sent directly to Spike Island, Barlow speculated that ‘the cellular discipline at Mountjoy would probably develop insanity’ and claimed that over the five years, 1.52 per cent of male prisoners and 2.59 per cent of female prisoners from Mountjoy were either sent to Dundrum or removed to Spike Island and noted as being insane on arrival.^94^ The figures reviewed by Barlow did not include weak-minded ‘ordinary’ prisoners, who, alongside ‘bodily invalids’ were often sent to Spike Island Prison. Dr O’Keeffe, who had worked at Spike Island prison for nearly 10 years prior to his appointment as surgeon at Mountjoy, claimed that during this time nearly all convicts—he was not referring to ‘ordinary’ prisoners—transferred from Spike Island to Drundrum Asylum had been in separation in Mountjoy. In 1895 the Departmental Committee on Prisons—the Gladstone Committee—which cast doubt on the efficacy of the highly centralised and disciplined system, implemented in the 1860s and 1870s, was defensive about claims that ‘the ratio of insanity in local prisons has exactly doubled since the introduction of a central system of administration’.^95^ In an memorandum on insanity in prisons, authored by Dr J. H. Bridges, it was acknowledged that ‘among the prison population the ratio of insanity arising among persons apparently sane on admission is not less than three times as great as that amongst the general population of corresponding ages’.^96^ It was therefore recommended that an additional medical appointment be made to the Prison Board of a candidate with expertise in treating mental disorders.^97^

While such rates prompted consternation among prison administrators and doctors alike, both groups were also eager to point out that these figures were complicated by the efforts of prisoners to feign insanity and the complexities of dealing with insanity in the prison system, which was increasingly depicted as gatekeeping crime and mental aberration. In 1896, Pentonville’s medical officer, Dr John Baker, was at pains to demonstrate that the number of insane in the prison system was exaggerated, claiming that ‘The form of insanity in many cases is conclusive evidence that mental defect existed before reception into prison’. He also asserted ‘that dangerous and insane criminals are being annually eliminated from the ranks of the community’. Baker, however, acknowledged that he had met with isolated cases where

there was reason to believe that the prisoners’ environment had adversely effected them. They embrace instances of melancholia induced by brooding over the barren vista of years to be spent in a long term of penal servitude, or of delusional insanity, probably due to the prolonged process of introspection, almost inseparable from cellular confinement.

‘These cases’, he concluded, ‘are few and far between’.^98^

The insistence that rates of mental illness in prisons were more closely related to the criminal justice system’s failure to identify and divert from prisons ‘real’ cases of insanity and ‘weak-mindedness’, alongside prisoners’ sustained efforts to feign mental illness, than to prison disciplinary regimes persisted. In 1905, David Nicolson, Superintendent of Broadmoor Criminal Lunatic Asylum between 1886 and 1896 and one of the Lord Chancellor’s Commissioners in Lunacy, along with Sir Christopher Nixon, Consulting Physician to Dundrum Asylum, and Irish Lunacy Inspector George P. O’Farrell, were appointed to devise ‘some general principles’ to assist prison and asylum medical officers in identifying ‘proper cases for treatment’ at Dundrum and to safeguard against the removal of prisoners feigning insanity to asylums.^99^ The committee also explored the problem of managing the ‘evolution of the weak-minded class in prison’ whose ‘moral obliquity, criminality, and general viciousness of conduct (*criminalmindedness*) form a combination which, in itself, cannot possibly be registered as insanity’.^100^ These prisoners were usually removed out of the stricter prison environments to invalid prisons, such as Woking Prison in England, established in 1858, but not to criminal lunatic asylums. In Ireland their numbers were considered too small to warrant the construction of a separate prison, and instead they were dispatched to Spike Island, and from the early 1880 s to Maryborough Prison.^101^ From their observations of these prisoners, medical officers such as Nicolson stressed that ‘although this condition is characterised by lowness of mental type, bluntness of moral sense, and stolidity and insensitiveness of nerve tone, it has none the less to be regarded as coming within the area of responsibility and punishability’. Such prisoners were to be retained within the prison albeit not subjected to its full rigours; they were not, however, to be removed to Broadmoor or Dundrum. As Nicolson, Nixon and O’Farrell argued: ‘The evolution of the weak-minded class of prisoner is brought about by the inherent resistive antagonism which dominates certain minds or types of mind, in the presence of penal discipline and restraint’. The activities of these prisoners—idleness, self-gratification and self-indulgence, malice and determination to escape the pains and penalties of prison life—revealed ‘sane motives’, and thus evidence of responsibility for their crimes. In such cases the ‘absence of well-established delusions and the predominance of insane-like, but not necessarily insane conduct … have the effect of causing some confusion as to the meaning or value of the term “insanity”’. ‘The extension of the area of sanity’, they argued, ‘so as to include prisoners for whom ordinary penal discipline has to be relaxed enables prisoners of this exceptional type to be detained in prison.’^102^

## Conclusion

Accurate figures for incidences of mental disorder in the prison system are not readily available, as official returns mainly focused on prisoners transferred to criminal and local asylums and, as this article has argued, these were only a subset of a larger group of prisoners who showed signs of mental disorder while confined. While prison administrators and medical officers in England and Ireland took care to present their institutions in the best possible light in official reports, their day-to-day records provide a more candid picture of incidences of mental disorder and of the mechanisms used to manage these cases. In their official reports and publications, they were likely to persist in playing down the effect of the prison regime—including separate confinement—in producing mental illness, arguing that the prison system picked up those who were ‘moral imbeciles’ and persistent offenders, prisoners with previous incidences of mental illness, and those whose criminal activities were related to and manifestations of their mental disease. These ‘lunatic convicts’ differed from ordinary lunatics, the ‘criminally insane’ and ordinary prisoners as they were more ‘cunning, deceitful, passionate, and impatient of control’ with a ‘naturally bad disposition complicated by a certain admixture of disease which tends to make the mind more fretful, irritable, and uncontrollable’.^103^ Medical officers and chaplains debated and developed these categories and labels within the prison walls in a very specific example of an assertion of ‘local power’ and expertise that emerged from the practicalities of managing their prisoner patients while simultaneously maintaining the order, discipline and reputation of their prisons and of the criminal justice system. The consequences for the prisoners could be bleak and harmful, as mental distress and desperation was punished and access to care delayed. While prison officials debated whether individual convicts were ‘truly’ insane, closely monitoring them for evidence of feigning, prisoners were left to languish within the harsh environment of prisons, and retained within a system that proved harmful to their mental well-being.

## Footnotes

1 The National Archive (TNA), PCOM 2/85, Pentonville Prison, Middlesex: Minute book, 1845–46, Meeting 31 January 1846, Medical Officer’s Journal, 160–1.

2
*Ibid*., 161–2; Fifth Report of the Commissioners for the Government of the Pentonville Prison (RCGPP), 1847 [818], ‘Fourth Yearly Report of the Physician’, 49.

3 TNA, PCOM 2/85, Pentonville Prison, Middlesex: Minute book, 1845–46, Meeting 14 February 1846, 175–6.

4
*Ibid*., Meeting 14 March 1846, 189.

5
*Ibid*., Meeting 25 April 1846, Medical Officer’s Journal, 220.

6 We focus here on male prisoners, who accounted for around four-fifths of the prison population in the second half of the nineteenth century; female prisoners were less often subjected to the full rigours of separate confinement, serving shorter terms of cellular isolation. For women in prison, see Lucia Zedner, *Women, Crime and Custody in Victorian England* (Oxford: Clarendon, 1991); Neil Davie, ‘“Business as Usual?” Britain’s First Women’s Convict Prison, Brixton 1853–1869, *Crime and Misdemeanours*, 2010, 4, 37–52; Beverly A. Smith, ‘The Female Prisoner in Ireland, 1855–1878’, *Federal Probation*, 1990, 54, 69–81.

7 Fifth RCGPP, 1847 [818], ‘Fourth Yearly Report of the Physician’, 49.

8 TNA, PCOM 2/85, Pentonville Prison, Middlesex: Minute book, 1845–46; TNA, PCOM 2/86, Minute book, 1846–47.

9 Ian O’Donnell, *Prisoners, Solitude and Time* (New York and Oxford: Oxford University Press, 2014), at 55.

10
*Ibid*., 42–3.

11 Michel Foucault, *Discipline and Punish: The Birth of the Prison*, translated from the French by Alan Sheridan (London: Allen Lane, 1977); Michael Ignatieff, *A Just Measure of Pain: The Penitentiary in the Industrial Revolution 1750–1850* (London and Basingstoke: Macmillan, 1978); David Garland, *Punishment and Modern Society. A Study in Social Theory* (Oxford: Clarendon, 1990).

12 Jean Daniel Jacob, Amélie Perron and Dave Holmes, eds, *Power and the Psychiatric Apparatus: Repression, Transformation and Assistance* (London: Routledge, 2014), 5.

13 Ignatieff, *A Just Measure of Pain*, 11.

14 Roy Porter, ‘Howard’s Beginning: Prisons, Disease, Hygiene’, in Richard Creese, W. F. Bynum and J. Bearn, eds, *The Health of Prisoners* (Amsterdam and Atlanta, GA: Rodopi, 1995), 5–26.

15 Miles Ogborn, ‘Discipline*,* Government and Law*:* Separate Confinement in the Prisons of England and Wales, 1830–1877’, *Transactions of the Institute of British Geographers*, 1995, 20, 295–311, at 296; Ignatieff, *A Just Measure of Pain*.

16 Bill Forsythe, ‘Russell, William Whitworth (1795–1847)’, *Oxford Dictionary of National Biography* (*DNB*), Oxford University Press, 2004; online edn, Jan 2008 <http://www.oxforddnb.com/view/article/73632>, accessed 15 December 2016; Bill Forsythe, ‘Crawford, William (1788–1847)’, *DNB*; online edn, Jan 2008 <http://www.oxforddnb.com/view/article/6646>, accessed 15 December 2016.

17 U. R. Q. Henriques, ‘The Rise and Decline of the Separate System of Prison Discipline’, *Past and Present*, 1972, 54, 61–93,at 79.

18 Under the silent system, associated in particular with Auburn Prison, New York, inmates were separated in cells for long periods during the day and worked and dined in association when silence was enforced through flogging. See William J. Forsythe, *The Reform of Prisoners, 1830–1900* (London and Sydney: St Martin’s Press, 1987), 15–29.

19 G. L. Chesterton, *Revelations of Prison Life*, 2 vols (London: Hurst & Blackett, 1856), vol. 2, 81, 80, 82.

20
*Ibid*., 14–15, 247–8.

21 ‘American Notes’, *The Times*, 25 October 1842. For the persistence of the system at the Eastern State Penitentiary, see A. T. Rubin, ‘A Neo-Institutional Account of Prison Diffusion’, *Law and Society Review*, 2015, 49, 365–99.

22 Inspectors of Prisons of Great Britain 1. Home District, Sixth Report, 1841 (147), IV.1, 228–9.

23
*The Times*, 24 November 1843.

24 Ignatieff, *A Just Measure of Pain*, 3–9.

25 Second RCGPP, 1844 [536], 18–20, Appendix B, No. 3 ‘Routine of a Day’, 190.

26 See Davie, ‘“Business as Usual?”’, 38.

27 Ignatieff, *A Just Measure of Pain*, 11. See Catherine Cox and Hilary Marland, ‘“He must die or go mad in this place”: Prisoners, Insanity and the Pentonville Model Prison Experiment, 1842–1852’, *Bulletin of the History of Medicine*, 2018, 92, 78–109 for more details of debates on the mental condition of prisoners in Pentonville.

28 See 5&6 Vict. 1842 c. 29, section xxiii; *The Times*, 1 May 1843, 24 November 1843.

29 Third Report of the Inspectors of Prisons of Great Britain, Part 1, 1837–38, 16.

30 John T. Burt, *Results of the System of Separate Confinement as Administered at the Pentonville Prison* (London: Longman, Brown, Green and Longmans; reprinted New York: Garland Publishing, 1984), 93.

31 Fifth RCGPP, 1847 [818], Report of the Commissioners, 12, Chaplain’s Report, 41–2.

32 TNA, PCOM 2/86, Pentonville Prison, Middlesex: Minute book, 1846–47, Meeting 26 September 1846, 33.

33 Eighth RCGPP, 1850 [1192], Chaplain’s Report, 16.

34 TNA, PCOM 2/85, Pentonville Prison, Middlesex: Minute book, 1845–46, Meeting 20 June 1846, 187.

35
*Ibid*., 267, 268.

36 TNA, PCOM 2/86, Pentonville Prison, Middlesex: Minute book, 1846–47, Meeting 26 September 1846, 37.

37
*Ibid*., Meeting 24 October 1846, 53, Meeting 5 December 1846, 110, 116.

38
*Ibid*., Visitors’ Minutes, 16 January 1847, 158. Clive Emsley, ‘Jebb, Sir Joshua (1793–1863)’, *DNB*, online edn Jan 2008 <http://www.oxforddnb.com/view/article/14683>, accessed 18 December 2016.

39 TNA, PCOM 2/86, Pentonville Prison, Middlesex: Minute book, 1846–47, Medical Officer’s Journal, 8 May 1847, 261.

40 TNA, PCOM 2/88, Pentonville Prison, Middlesex: Minute book, 1848–49, Meeting 24 February 1849, 145–6.

41 See Cox and Marland, ‘“He must die or go mad in this place”’.

42 ‘The Medical Society of London’, *Lancet*, 1 November 1850, 535.

43 Peter Laurie, *“Killing no Murder;” or the Effects of Separate Confinement on the Bodily and Mental Condition of Prisoners in the Government Prisons and other Gaols in Great Britain and America* (London: John Murray, 1846).

44 Fifth RCGPP, 1847 [818], Report of the Commissioners, 41–2; National Archives of Ireland (NAI), Government Prisons Office (GPO)/PN/4-5, Philipstown Character Books, 1847–62.

45 Fifth RCGPP, 1847 [818], Report of the Commissioners, 11–12.

46 Report on the Discipline and Management of the Convict Prisons (RDMCP), 1851 [1572], Pentonville Prison, 9–10.

47 TNA, PCOM 2/86, Pentonville Prison, Middlesex: Minute book, 1846–47, Visitors’ Minutes, 16 January 1847, 158–9.

48 Report from the Select Committee on Prison Discipline; together with the Proceedings of the Committee, Minutes of Evidence, Appendix and Index, 1850 (632), iv–vi, at iv.

49 Ogborn, ‘Discipline, Government and Law’, 304; see also Forsythe, ‘Centralisation and Autonomy: The Experience of English Prisons 1820–1877’, *Journal of Historical Sociology*, 1991, 4, 317–45.

50 Ignatieff, *A Just Measure of Pain*, 197.

51 Liverpool Record Office (LRO), 347 MAG/1/2/1, Minutes of the Quarterly and Annual Meetings of the Visiting Justices of the Borough Gaol and House of Correction, also Special Gaol Sessions, 1852–1864, 27 October 1855; *ibid*., 20 May 1857. Occasionally prison staff brought in an additional prisoner to share a cell and watch a prisoner regarded as depressed in mind and at risk of self-harm or suicide. LRO, 347 JUS 4/1/2, Minutes of Justices Sessions Gaol and House of Correction, October 1864–1870, 25 July 1867, 125.

52 Prisons (Separate Confinement), HC 1856 (163), xlix, 1–9.

53 Berkshire Record Office (BRO), Q/S0 24, Sessions Order Book April 1853–July 1855, General Quarter Sessions, 3 January 1853, Surgeon’s Report, 644.

54
*Ibid*., Chaplain’s Report, General Quarter Sessions, 17 October 1853, 143.

55 Wakefield Record Office Q/S 10/56, Quarter Sessions Order Book, October 1846–April 1850, 98; RDMCP, 1852 [1659], Report of Lieut. Col. Jebb, 9.

56 RDMCP, 1852 [1659], Report of Lieut. Col. Jebb, 5.

57 Patrick Carroll-Burke, *Colonial Discipline. The Making of the Irish Convict System* (Dublin: Four Courts Press, 2000), 56–7.

58 NAI, GPO/Letter Book (LB)/12, Letter from Henry M. Hitchins to Francis Rynd, 14 February 1850, 35.

59
*Ibid*., Letter from Henry M. Hitchins to unknown, 12 February 1850, 53.

60
*Ibid*., Letter from Henry M. Hitchins to Major Thomas Reddington, 22 March 1850, 63.

61 Copies of Correspondence Relative to the Management and Discipline of Convict Prisons, and the Extension of Prison Accommodation, with Reports of Commissioners (Ireland), 1854 (344), 16.

62
*Ibid*., 18; Carroll-Burke, *Colonial Discipline*, 95. The 1854 inquiry recommended the ‘overhaul’ of the convict prisons prompting the establishment of the Directors of Convict Prisons for Ireland in 1854 with Walter Crofton as Chair, see Carroll-Burke, *Colonial Discipline*, 99–100.

63 Copies of Correspondence Relative to the Management and Discipline of Convict Prisons (Ireland), 1854 (344), 18.

64 First Annual Report of the Directors of Convict Prisons in Ireland, for the Year ended 31st December, 1854; with Appendix, H.C. 1854–55 [1958], xxvi, 39.

65 Burt, *Results of the System of Separate Confinement*, preface, ix–x.

66
*Ibid*., 89.

67
*Ibid*., 91.

68
*Ibid*., 106.

69 Twenty-First Report of the Inspectors Appointed to Visit the Different Prisons of Great Britain. Northern and Eastern District, 1857 [2250], 24, 54.

70 LRO, 347 MAG 1/3/3 Proceedings of the Meetings of the Visiting Committee, April 1878–June 1897, 84, 96, 116, 152, 153, 224, 263; LRO, M614 RAI/8/16 Rainhill Asylum Female Casebook, July 1892–March 1894, 178; LRO, M614 RAI/8/18 Rainhill Asylum Female Casebook, October 1895–July 1897, 159.

71 NAI, GPO/PN/4 Philipstown Character Books, 1847–62, Reg. no.1742.

72 Martin J. Weiner, ‘The Health of Prisoners and the Two Faces of Benthamism’, in Creese, Bynum and Bearn, eds, *The Health of Prisoners*, 44–58, at 47–8.

73 Joe Sim, *Medical Power in Prisons: The Prison Medical Service in England 1774–1989* (Milton Keynes and Philadelphia, PA: Open University Press, 1990).

74 Weiner, ‘The Health of Prisoners’, 52–3.

75 Select Committee of House of Lords on State of Prison Discipline in Gaols and Houses of Correction [Carnarvon Committee], 1863 (499), 370.

76 Reports of the Directors of Convict Prisons on the Discipline and Management of Pentonville, Millbank and Parkhurst Prisons, and of Portland, Portsmouth, Dartmoor, Chatham and Brixton Prisons, with Fulham Refuge and the Invalid Prisons at Lewes and Woking, for the year 1859, 1860 [2713], 67.

77
*Ibid*., 74.

78 NAI, GPO/Correspondence (CORR)/1860/257/Mountjoy, Letter from Robert McDonnell to Governor, 15 December 1860.

79 NAI, GPO/CORR/1860/6/Mountjoy, Letter from Robert McDonnell to Governor, 28 January 1860.

80 Bill Forsythe, ‘Loneliness and Cellular Confinement in English Prisons 1878–1921’, *British Journal of Criminology*, 2004, 44, 759–70.

81 Thomas Laycock, ‘Suggestions for Rendering Medico-Mental Science Available to the Better Administration of Justice and the more Effectual Prevention of Lunacy and Crime’, *Journal of Mental Science*, 1886, 14:67, 334–45, at 334–5.

82 William Forsythe, *Penal Discipline, Reformatory Projects and the English Prison Commission 1895–1939* (Exeter: University of Exeter Press, 1990), 20. Du Cane was in correspondence with the statistician and anthropologist, Francis Galton, see Neil Davie, *Tracing the Criminal. The Rise of Scientific Criminology in 1860–1918* (Oxford: The Bardwell Press, 2005), 96. See Bill Forsythe, ‘Du Cane, Sir Edmund Frederick (1830–1903)’, *DNB*; online edn, Jan 2008 <[http://www.oxforddnb.com/view/article/32910>, accessed 20 December 2016.

83 John Campbell, *Thirty Years Experience of a Medical Officer in the English Convict Service* (London: T. Nelson and Sons, 1884), 65, 70.

84 NAI, GPB/CORR/1888/1365, Papers relating to padded cell at Castlebar Prison, 1886–88.

85 Arthur Griffiths, *Memorials of Millbank, and Chapters in Prison History* (London: Henry S. King, 1875), 177–8, 180.

86 Campbell, *Thirty Years Experience*, 78–9.

87 ‘Criminal Lunatics and Lunatic Convicts’, *British Medical Journal*, 4 July 1874, 14–15, at 15.

88 There is an expansive literature on moral management. See e.g. Andrew Scull, *The Most Solitary of Afflictions: Madness and Society in Britain, 1700–1900* (New Haven, CT and London: Yale University Press, 1993), ch. 4. For a comparison of the therapeutic regimes at Broadmoor criminal asylum for different classes of inmate, see Jade Shepherd, ‘“I am very glad and cheered when I hear the flute”: The Treatment of Criminal Lunatics in Late Victorian Broadmoor’, *Medical History*, 2016, 60, 473–91.

89 NAI, GPO/ LB/15, C.R. Knight to Local Inspector, Spike Island, 19 January 1856; *ibid*., Walter Crofton to the Inspectors of Lunatic Asylums, 26 January 1856; NAI, General Prison Board (GPB)/Minute Book (MB)/3, 22 January 1885, 187.

90 NAI, GPB/CORR/1888/3881, Correspondence relating to inquest on Catherine Kelly, March–April 1888. Dr James Ridley committed suicide in July 1888 during the inquest into the death of the nationalist campaigner, John Mandeville, who had been released from Tullamore prison before Christmas 1887. His family and supporters claimed his treatment in prison had caused his death. See Beverly A. Smith, ‘Irish Prison Doctors. Men in the Middle, 1865–90’, *Medical History*, 1982, 26, 371–94.

91 TNA, HO 144/469/X6313. Lunacy: Removal to asylum of prisoners certified insane, 1885.

92 TNA, HO 114/513/X66658. Lunacy: Edward Cox. Injuries to insane prisoner inquiry, 1897–1898.

93 Edwin Pears, *Prisons and Reformatories at Home and Abroad, being the Transactions of the International Penitentiary Congress held in London, July 3–13, 1872, including Official Documents, Discussions, and Papers Presented to the Congress.* An Account of the Manner in which Sentences of Penal Servitude are Carried out in England by Sir Edmund Frederick Du Cane (London: Longman’s, Green, and Co., 1872), 305.

94 Royal Commission on Prisons in Ireland. Vol. I. Reports, with Digest of Evidence, Appendices, 1884–85 [C.4233] [C.4233-I], 277.

95 Prisons Committee. Report from the Departmental Committee on Prisons, 1895 [C.7702] [C.7702-I], 7. See Forsythe, *Penal Discipline* for a detailed assessment of the Gladstone Committee.

96 Prisons Committee. Report from the Departmental Committee on Prisons, 1895 [C.7702] [C.7702-I], 290–1.

97
*Ibid*., 34.

98 John Baker, ‘Insanity in English Local Prisons, 1894–95’, *Journal of Mental Science,* 1896, 42, 294–302, on pp. 295, 301, 302.

99 NAI, Chief Secretary’s Office, Registered Papers (CSORP)/1905/12904, Minute from George Plunkett O’Farrell and E. M. Courtney, Office of the Inspectors of Lunatic Asylums, to Under Secretary, 10 June 1904; James Crichton-Browne, ‘David Nicolson’, *Journal of Mental* Science, 1933, 79, 1–3; Helen Andrews, ‘Nixon, Sir Christopher John (1849–1914)’, in James McGuire and James Quinn eds, *Dictionary of Irish Biography* (Cambridge: Cambridge University Press, 2009); online edn, <[http://dib.cambridge.org.ucd.idm.oclc.org/advancedsearch.do#>, accessed 18 December 2016.

100 NAI, CSORP/1905/12904, Report of the Committee of Inquiry into Certain Doubtful Cases of Insanity amongst Convicts and Persons Detained during the Pleasure of the Lord Lieutenant to his Excellency William Humble, Earl of Dudley (1905), 10. For more on the attitudes towards ‘weak-minded’ prisoners, see Stephen Watson, ‘Malingerers, the Weakminded Criminal and the “Moral Imbecile”: How the English Prison Medical Officer became an Expert in Mental Deficiency, 1880–1930’, in Michael Clark and Catherine Crawford, eds, *Legal Medicine in History* (Cambridge: Cambridge University Press, 1994), 223–41, at 226–7.

101 Fifth Report of the General Prisons Board, Ireland, 1882–83; with Appendices, 1883 [C.3757], 5. For the relationship between crime, degeneration and biological inferiority, see Davie, *Tracing the Criminal* and Nicole Hahn Rafter, *Creating Born Criminals* (Urbana, IL: University of Illinois Press, 1997).

102 NAI, CSORP/1905/12904, Report of the Committee of Inquiry into Certain Doubtful Cases of Insanity amongst Convicts, June 1905, 10.

103 NAI, GPO/CORR/1860/6/Mountjoy, Letter from Robert McDonnell to Governor, 28 January 1860.

## Notes

Associate Professor Catherine Cox is co-Principal Investigator on the Wellcome Trust Investigator Award, ‘Prisoners, Medical Care and Entitlement to Health in England and Ireland, 1850–2000’, working on mental health in prison. She is author of *Negotiating Insanity in the Southeast of Ireland, 1820–1900* (2012) and has published on mental health and migration, institutionalisation and nineteenth-century medical practice in Ireland.

## Notes

Professor Hilary Marland is co-Principal Investigator on the Wellcome Trust Investigator Award, ‘Prisoners, Medical Care and Entitlement to Health in England and Ireland, 1850–2000’. Her strand of research focuses on mental health in prison. She is author of *Dangerous Motherhood: Insanity and Childbirth in Victorian Britain* (2004) and *Health and Girlhood in Britain, 1874–1920* (2013), and has also published on migration and mental illness, nineteenth-century medical practice, midwifery and obstetrics, and domestic practices of medicine.

